# Telomerase Contributes to Fludarabine Resistance in Primary Human Leukemic Lymphocytes

**DOI:** 10.1371/journal.pone.0070428

**Published:** 2013-07-29

**Authors:** May Shawi, Tsz Wai Chu, Veronica Martinez-Marignac, Y. Yu, Sergei M. Gryaznov, James B. Johnston, Susan P. Lees-Miller, Sarit E. Assouline, Chantal Autexier, Raquel Aloyz

**Affiliations:** 1 Department of Medicine, Division of Experimental Medicine, McGill University, Montreal, Quebec, Canada; 2 Department of Anatomy and Cell Biology, McGill University, Montreal, Quebec, Canada; 3 Oncology Department, McGill University, Montreal, Quebec, Canada; 4 Bloomfield Centre for Research in Ageing, Jewish General Hospital, Montreal, Quebec, Canada; 5 Lady Davis Institute for Medical Research & Cancer Segal Center, Jewish General Hospital, Montreal, Quebec, Canada; 6 Geron Corporation, Menlo Park, California, United States of America; 7 Manitoba Institute of Cell Biology, Cancer Care Manitoba, Winnipeg, Manitoba, Canada; 8 University of Calgary, Department of Biochemistry and Molecular Biology, Southern Alberta Cancer Research Institute, Calgary, Alberta, Canada; University of Kansas Medical Center, United States of America

## Abstract

We report that Imetelstat, a telomerase inhibitor that binds to the RNA component of telomerase (hTR), can sensitize primary CLL lymphocytes to fludarabine *in vitro*. This effect was observed in lymphocytes from clinically resistant cases and with cytogenetic abnormalities associated with bad prognosis. Imetelstat mediated-sensitization to fludarabine was not associated with telomerase activity, but with the basal expression of Ku80. Since both Imetelstat and Ku80 bind hTR, we assessed 1) if Ku80 and Imetelstat alter each other's binding to hTR in vitro and 2) the effect of an oligonucleotide complementary to the Ku binding site in hTR (Ku oligo) on the survival of primary CLL lymphocytes exposed to fludarabine. We show that Imetelstat interferes with the binding of Ku70/80 (Ku) to hTR and that the Ku oligo can sensitize CLL lymphocytes to FLU. Our results suggest that Ku binding to hTR may contribute to fludarabine resistance in CLL lmphocytes. This is the first report highlighting the potentially broad effectiveness of Imetelstat in CLL, and the potential biological and clinical implications of a functional interaction between Ku and hTR in primary human cancer cells.

## Introduction

B-cell chronic lymphocytic leukemia (CLL) is a biologically and clinically heterogeneous incurable disease [1]. CLL is characterized by the accumulation of clonal malignant B lymphocytes in the blood of affected patients primarily due to decreased apoptosis [2]. In CLL several biological traits have been associated with bad prognosis including short telomeres, high telomerase expression or activity and limited mutation in the IgVH locus (unmutated IgVH) [3], [4], [5], [6], [7], [8]. In humans, telomerase consists of a catalytic reverse transcriptase (RT) subunit (hTERT) and an RNA subunit (hTR) that serves as a template for telomere synthesis [9]. The cytotoxic effect of telomerase inhibition in dividing human malignant cells is associated with impaired telomere length maintenance [10], [11]. By uncharacterized mechanisms independent of telomerase's role in telomere length maintenance, telomerase inhibition can also decrease the DNA repair capacity of cancer cells and potentiatethe cytotoxic effect of anticancer agents [10], [12], [13]. Athough a proliferating compartment exists in CLL (i.e. in lymphatic nodes and bone marrow), most of the circulating CLL lymphocytes are quiescent [14]. Standard treatment of CLL is based on fludarabine (FLU) [15], [16]. FLU is a purine nucleoside analogue that is toxic to non cycling cells by induction of DNA double strand breaks (DSBs) [17], [18], [19]. FLU-induced DNA damage is repaired by non homologous end joining (NHEJ) [20]. NHEJ-mediated DNA repair is initiated by the recruitment of the DNA-dependent protein kinase catalytic subunit (DNA-PKcs) to damaged DNA. Recruitment of DNA-PKcs and other NHEJ factors (i.e. XLF and XRCC4-Ligase IV) is mediated by the Ku70/80 (Ku) heterodimer [21]. In addition, Ku has been reported to interact with the telomerase RNA subunit, hTR [22], [23]. In most organisms examined, mutations in either Ku70 or Ku80 result in the expected deficits in DNA DSB repair, but in human cells a Ku deletion (but not a DNA-PKcs deletion) is lethal [24], [25]. Loss of Ku expression in human cells results in a rapid loss of cell viability, increased apoptosis, significant loss of telomeric sequences and increased chromosomal aberrations [24], [25]. Ku mRNA levels in CLL lymphocytes are lower than in normal B cells [26].

Although telomerase activity is a prognostic factor in CLL, whether telomerase contributes to drug resistance or is instead a marker of aggressive activated clones is not known. Moreover, understanding the potential role of telomerase in largely nonproliferative cells is also of importance. This prompted us to assess the effects of a telomerase inhibitor, Imetelstat (a.k.a. GRN163L), on the *in vitro* response to FLU of primary lymphocytes isolated from peripheral blood of CLL patients. Imetelstat is a lipid-modified N3′–>P5′ thio-phosphoramidate oligonucleotide that targets the telomerase RNA template [27]. The U.S. National Institutes of Health reports thirteen clinical trials assessing Imetelstat in solid tumors and hematological malignancies, including CLL (clinicaltrials.gov; 2009). We identify Imetelstat as a potential combination therapy with DNA damaging chemotherapeutics and provide evidence that *in vitro* efficacy is associated with the basal expression of Ku. This is the first report highlighting potential biological and clinical implications of a functional interaction between Ku and hTR in primary human cancer cells.

## Materials and Methods

### Patients

Forty four patients diagnosed with B-CLL were enrolled in this study after written informed consent at the Jewish General Hospital in Montreal with the approval of the Institutional Ethics Committee.

### Purification, isolation and characterization of CLL lymphocytes

Lymphocytes were purified from peripheral blood using Ficoll-hypaque. Expression of CD5, CD19 and CD38 was assessed by flow cytometry. Only samples with <5% T lymphocyte (CD4^+^) contamination were utilized. IgVH status was assessed using standard procedures [28].

### Compounds used

We tested the following compounds: FLU (Sigma-Aldrich Co., St Louis, MO, USA), Imetelstat [27], a mismatch oligonucleotide [29], a sense oligonucleotide fully complementary to Imetelstat (Geron Corporation, Menlo Park, CA, USA) [27] and BIBR1532 (Boehringer Ingelheim, Vienna, Austria). In addition we tested a lipidated oligonucleotide complementary to the Ku binding region in hTR (nucleotides 404–417) (Ku oligo hereafter) (selected after a preliminary screening using eight CLL samples and four different non-lipidated oligonucleotides complementary to hTR) (**Table S2**). Specifically, the Ku oligo was selected based on an initial screening to assess the effect of various oligonucleotides on FLU-induced cytotoxicity (Data not shown).

### Sensitization Assessment

Cytotoxicity was determined using the colorimetric MTT assay 72****hours after *in vitro* treatment [28]. The IC_50_s were calculated by interpolation as the concentration of FLU which results in an OD equivalent to 50% of the OD obtained in cells treated with vehicle dimethylformamide (DMF). Briefly, the lymphocytes were treated with FLU (0–30 µM) alone or in combination with 1 µM of Imetelstat, 1 µM Ku oligo or 2.5 µM BIBR1532. The synergy or sensitization (R value) was determined as the ratio between the IC_50_ of FLU alone/IC_50_ of FLU in the combinations. When R>1, the interaction is synergistic, when R = 1, there is no synergy, and when R<1 there is an antagonistic interaction [18].

### Telomerase Activity

To assess telomerase activity, the “Telo TAGGG telomerase PCR Elisa” kit was used (Roche Diagnostics, Mannheim, Germany) following the manufacturer's instructions. This assay is a photometric enzyme immunoassay for quantitative determination of telomerase activity, utilizing the Telomeric Repeat Amplification Protocol (TRAP) commonly used to estimate telomerase activity in primary CLL lymphocytes [3], [5].

### Western blotting

Proteins extracted were prepared as described [28]. Briefly, 50 µg of protein extracts were resolved on 4–20% gradient SDS-PAGE gels and transferred onto nitrocellulose membranes (BioRad Laboratories, Hercules, CA, USA), probed with specific antibodies (α-actin, from Neomarkers, α-Ku80 and α-H2AX from Cell Signaling) and developed using horseradish peroxidase secondary antibodies and chemiluminescence solution (GE Healthcare Bio-Sciences Corp., Piscataway, NJ, USA). After exposure, the X-ray films were scanned and the signals quantified using the software NIH Scion Images. Equal protein loading was confirmed by reprobing as indicated [28].

### Synthesis and labeling of the human telomerase RNA and Imetelstat

hTR synthesis and 5′-end labeling was performed as previously described [30]. 5′- end labeling of Imetelstat was performed similarly to 5′end labeling of hTR.

### Electrophoretic mobility shift assays (EMSA)

Binding reactions were performed as previously described [23]. Briefly, 0.2 µg of purified Ku70/80 [31] was incubated with labeled hTR (2 µl at 2500 cpm/μl) in 10 µl containing 100****mM NaCl, 1****mM MgCl_2_, 10****mM HEPES pH 7.5, 5% (w/v) glycerol, 0.8 unit of RNase OUT and varying concentrations of Imetelstat or control sense oligonucleotide. The reaction mixtures were incubated at 25°C for 10****min and electrophoresed through a composite gel system [32]. Binding reactions between Ku and labeled Imetelstat were performed as follows. Increasing amounts of Ku (0 µg, 0.2 μg or 0.6 µg) were incubated with 0.02 pmol of hTR and 250 pM of labeled Imetelstat in a final volume of 10 µl.

#### Flow Cytometry Analysis

After fixation with 1% paraformaldehyde at 4°C for 15****min, cells were washed with PBS and then permeabilized in 70% cold ethanol for 20****min in ice. After washing in PBS, the cells were incubated 30****min at room temperature in dilution buffer (PBS, 1% bovine serum albumin, and 0.1% Triton X-100), and then they were incubated overnight at 4°C with a rabbit anti-phosphorylated DNA-PK (S2056) antibody (Abcam, Hornby, ON, Canada) 1∶200 in dilution buffer. The cells were washed in PBS, then incubated for 60****min with a goat anti-rabbit Alexa Fluor 488 secondary antibody (Molecular Probes) diluted 1∶500 in dilution buffer, washed with PBS, stained overnight at 4°C with 7-aminoactinomycin D (BD Biosciences PharMingen, San Diego, CA), and then analyzed by flow cytometry as described previously [33].

### Statistical analysis

Differences between groups were assessed using the paired t-test, t-test, Mann–Whitney test and ANOVA as indicated. Two-sided tests with an α-value of 0.05 were used. Correlations between the data were performed using the non-parametric Spearman rank correlation analysis, Pearson correlation or linear regression assessment with an α-value of 0.05 as indicated. Non-parametric analysis was performed by Chi-square analysis or Fisher test using as a cut off the median values for the parameters analyzed except for IgVH status, telomerase activity and clinical status. All tests were performed using Sigmastat software (Systat Software Inc., San Jose, CA, USA). A p-value lower than 0.05 was considered to be statistically significant. Unless otherwise indicated in the legends to the figures, the boxes represent the 25^th^/75^th^ percentiles and the line in the box represents the median value of the variables. The top whisker represents the values associated with the 75^th^ to 90^th^ percentiles, and the bottom one represents the values associated with the 25^th^ to 10^th^ percentiles.

## Results

**Figure 1:**
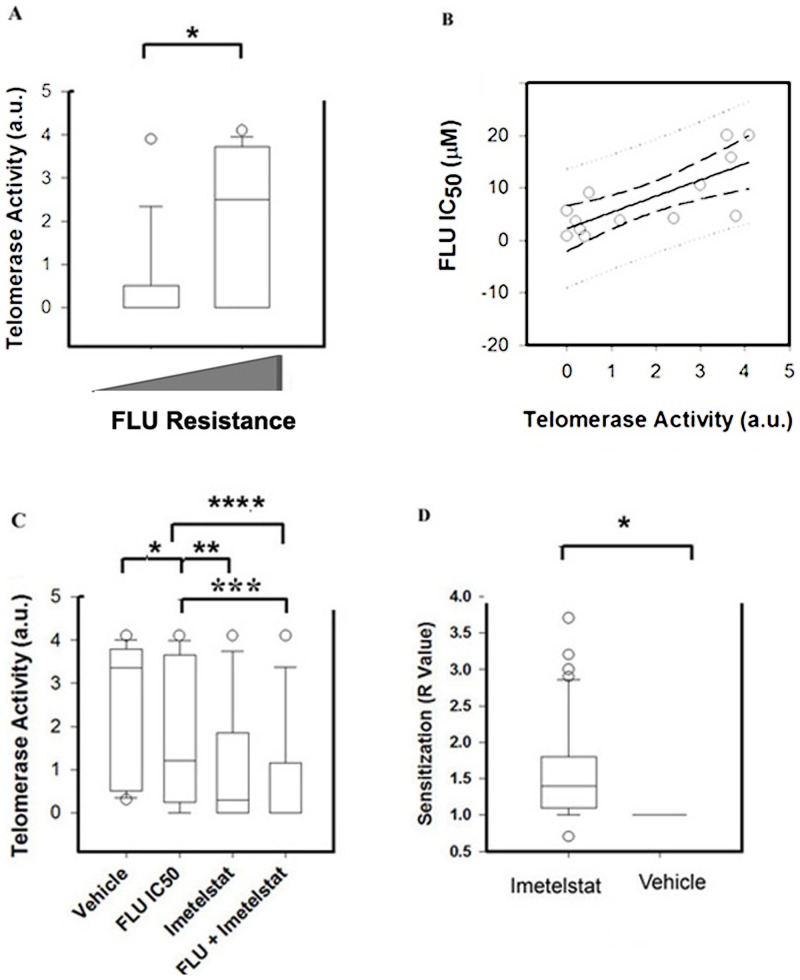
Telomerase Activity is associated with *in vitro* FLU resistance. A) Basal telomerase activity (y-axis) was lower in FLU sensitive CLL lymphocyte samples (left bar) compared to FLU resistant CLL lymphocyte samples (right bar) (Mann-Whitney Rank Sum Test, *p = 0.02). The cut off to define sensitivity or resistance to FLU was 2.5 µM, the median FLU IC_50_. B) FLU resistance of lymphocytes from CLL patients (y-axis) was associated with telomerase activity measured after FLU treatment (x-axis). Pearson Product Moment Correlation coefficient of r = 0.75 with a value p = 0.003. The solid line is the regression line. The straight dotted line represents the confidence interval of the population and the curved dotted line the confidence interval of the regression. C) Effect on telomerase activity (y-axis) of the indicated treatments (x-axis). Kruskal-Wallis One Way Analysis of Variance on Ranks indicates that the treatments significantly affected telomerase activity (****p = 0.007) followed by Wilcoxon Signed Rank test for paired samples (p =  *0.006_;_ **p = <0.001 and ***P = 0.002). D) Imetelstat significantly sensitized primary CLL lymphocytes to FLU. The R value obtained with Imetelstat was significantly different from 1, Wilcoxon Signed Rank test of paired samples indicates a significant difference (*p<0.001).

The characteristics of the 44 CLL cases studied are shown in **Table S1**. Twenty six patients were clinically untreated, twenty patients were female, the median age was 68****years old (ranging from 44 to 89 years) and twenty cases presented unmutated IgVH (U-IgVH hereafter) (<2% mutated with respect to germ line). In addition, all four Rai stages were represented in the panel of samples used (3 cases with Rai O, 12 cases with Rai I, 10 cases with Rai II, 5 cases with Rai III and 11 cases with Rai IV), where higher Rai stage represents more advanced disease. The CLL sample panel includes four patient samples positive for del11q (ATM locus) and three patient samples positive for del17p (TP53 locus) (>20% of positive cells). One del17 case was assessed before and after clinical treatment (#19 and #19a respectively). FLU IC_50_s were obtained using the MTT assay. This assay is routinely used to assess cytotoxicity in primary CLL lymphocytes in vitro [34] and IC_50_s obtained using this assay have been shown to correlate with changes in lymphocyte counts and with clinical response of CLL patients [35], [36]. The median value of *in vitro* FLU IC_50_ in lymphocytes from CLL patients was 2.5 µM (ranging from 0.6 to 20 µM (**Table S1**). The *in vitro* FLU IC_50_s were significantly higher in the lymphocytes from clinically resistant (CR) patients (ANOVA p<0.05) with median FLU IC_50_ values of 14.4 µM in clinically resistant patient samples versus 1.7 and 2 µM in clinically untreated (U) and clinically treated (T) cases respectively (**Figure S1 A**). The cases were considered clinically resistant if the patients relapsed within six months of the last treatment. As previously reported [5], [8], [37], telomerase activity was associated with U-IgVH status (Chi-square analysis p<0.0001). Eighty-six percent of the U-IgVH cases were telomerase positive while only fourteen percent of the M-IgVH cases were telomerase positive. In addition, FLU resistance of lymphocytes from CLL patients was strongly associated with both basal telomerase activity (**Figure 1A**), telomerase activity after FLU treatment (Pearson correlation, r = 0.75, p = 0.003) but not with Ku80 expression **(** **Figure 1B** **)**.

### Imetelstat sensitizes primary CLL lymphocytes to FLU, including patient samples from del11q and del17p positive cases

**Figure 2:**
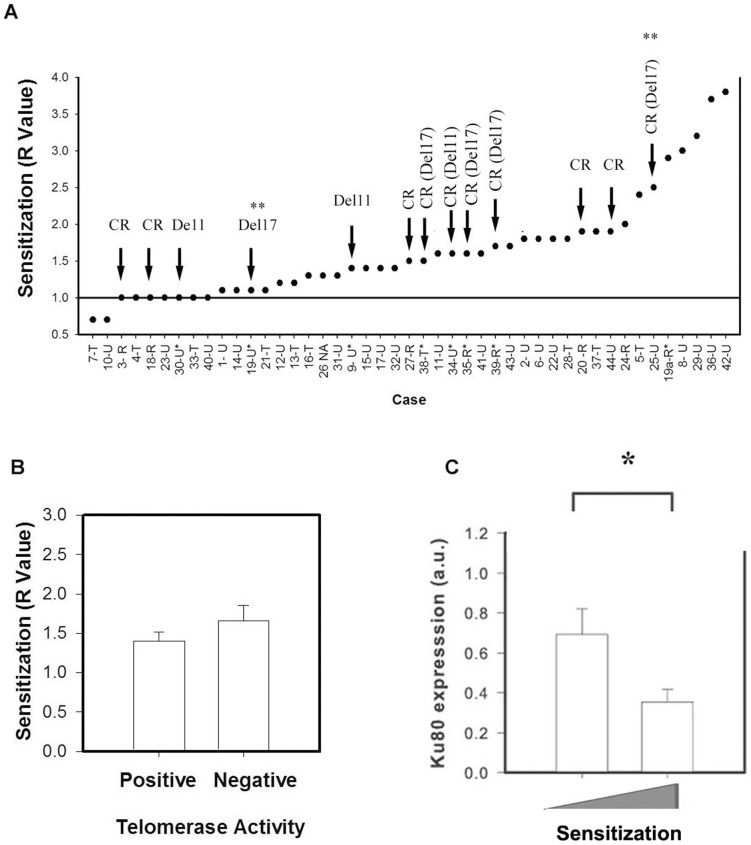
Imetelstat-mediated sensitization to FLU is associated with Ku expression. A) The plot represents the sensitization values obtained (y-axis) for CLL lymphocytes from each case (x-axis). The arrows indicate the clinically resistant (CR) cases and cases positive for del11 and del17. The ** indicated a del17 case that changed from untreated to clinically resistant with increased percentage of cells positive for del17 (Table S1). B) Comparison of the sensitization R values (y-axis) between telomerase negative (right bar) and telomerase positive samples (left bar) (Mann-Whitney Rank Sum Test p = 0.295). C) Ku80 expression (y-axis) in primary CLL lymphocytes samples segregated by the median R value (1.5) (Mann-Whitney Rank Sum Test *p = 0.03).

We next assessed the cytotoxicity of Imetelstat alone or in combination with FLU *in vitro*. We selected 1 µM Imetelstat because the reported Imetelstat Cmax ranges from 1.4 to 4 µM) [38]. We found that this concentration of Imetelstat significantly decreased telomerase activity in both vehicle (3.5 a.u. median value) and FLU (1.0 a.u. median value) treated samples to undetectable levels (ANOVA, p = 0.007 followed by t-test p<0.002) **(** **Figure 1C** **)**. Imetelstat alone was not cytotoxic to CLL lymphocytes. However, 1 µM Imetelstat significantly sensitized the CLL samples tested to FLU (**Table S1** and **Figure 1D**) (p<0.001). Sensitization is defined by the ratio of the IC_50_ of FLU plus vehicle to the IC_50_ of FLU plus 1 µM Imetelstat (R value). The R values obtained ranged from 0.7 to 3.8 with a median value of 1.5. Sensitization (R>1) was observed in thirty six patient samples. Antagonism was observed in two patient samples (R = 0.7) and no effect of Imetelstat on FLU sensitivity was observed in seven patient samples (R = 1) (**Table S1**). In addition, Imetelstat sensitized CLL lymphocytes to FLU in five of seven CLL samples from clinically resistant patients, three of four del11 patient samples and all three del17 patient samples. Sensitization to FLU by Imetelstat was observed in CLL lymphocytes from the del17 case 19 after development of clinical resistance (case 19a) (**Figure 2A**). No statistical difference was observed in the median value of Imetelstat-mediated sensitization to FLU between del11 and del17 samples and the rest of the samples tested (1.55 *vs.* 1.4 respectively p>0.05). Proficiency of ATM signaling was assessed in all del11 patient samples (cases 9, 30, 34 and 35) by monitoring p53 and p21 protein levels forty eight hours after FLU treatment *in vitro* as previously described [39]. FLU treatment failed to increase p53 and p21 expression only in CLL lymphocytes from case 34, indicating that in this case the remaining ATM allele is mutated (data not shown). However, CLL lymphocytes from this case were sensitized to FLU by Imetelstat (R value  = 1.6). As controls we used 1 µM of either a scrambled sequence oligonucleotide, or a sense oligonucleotide, which does not inhibit telomerase activity [29] and did not have an effect on FLU sensitivity (data not shown).

### Imetelstat sensitization to fludarabine is associated with Ku expression

Overall, the sensitization values we obtained (R) were not associated with clinical status, Rai Stage or prognostic factors in CLL such as CD38 expression [40], IgVH mutational status, telomere lengh (p>0.05, data not shown) [3], [4], [5], [6], [7], [8]. Imetelstat-mediated sensitization to FLU was not associated with either FLU resistance or the effect of Imetelstat on telomerase activity. In contrast, basal Ku80 expression was inversely associated with Imetelstat-mediated sensitization to FLU (i.e. better sensitization in samples expressing lower levels of Ku80). This association was significant using two non parametric tests (Spearman Correlation r = −0.48, p = 0.01 2 and Chi Square test p = 0.036). In addition when the telomerase activity and Ku expression values in the samples tested were segregated by the median value of Imetelstat-mediated sensitization to FLU, there was no difference in telomerase activity (**Figure 2B**) but a two-fold difference in the basal expression of Ku80 (**Figure 2C**) (p = 0.03). This association was stronger in the subset of samples with detectable basal telomerase activity (Spearman Rank Correlation, r = −0.76, p = 0.0001, data not shown). A representative western blot of Ku80 levels in CLL samples is shown in **Figure S1B**. Importantly there was an 18-fold difference in the expression of Ku80 among the samples tested, ranging from 0.1 to 1.8 (a.u.).

### Imetelstat interferes with the binding of Ku to hTR *in vitro*

**Figure 3:**
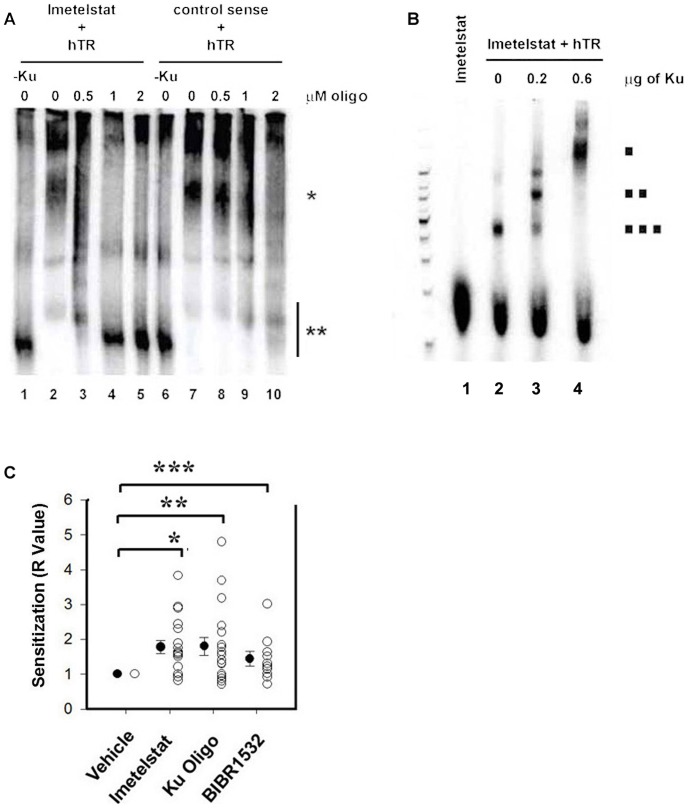
A catalytic site inhibitor of telomerase or an oligonucleotide targeting the Ku binding site of hTR sensitize CLL lymphocytes to FLU. A) Formation of the Ku70/80-hTR complex is completely inhibited by 1 µM Imetelstat (n = 3) but not by the control sense oligonucleotide (n = 2). Lane 1: radiolabeled hTR (denoted by **). Lane 2: The formation of a Ku70/80-hTR complex (denoted by *) occurs upon the addition of 0.2ug Ku70/80. Increasing concentrations of 0.5, 1 and 2 µM Imetelstat (lanes 3–5) or a control sense oligonucleotide (lanes 8–10) were added. B) Addition of Ku resulted in supershifts of the hTR-Imetelstat complex. Lane 1: radiolabeled Imetelstat. Lane 2: The formation of hTR-Imetelstat complex occurs upon addition of 0.02 pmol hTR (denoted by 3 squares). Lanes 3 and 4: Addition of 0.2 or 0.6 µg Ku results in supershift of the hTR-Imetelstat complex (denoted by 1–2 squares). C) The dot plot represents the sensitization R values (y axis) obtained after the indicated treatments (x axis). The open circles represent the values obtained in each sampe and the closed circles represent the mean value of sensitization in each group and the whiskers the standard error. Kruskal-Wallis One Way Analysis of Variance on Ranks (****p = 0.007) followed by Wilcoxon Signed Rank Test ***P = <0.001, Paired t-test **p = 0.001 and *p<0.05.

Our results suggest that Imetelstat interferes with Ku-associated pro-survival processes that contribute to FLU resistance. Since both Ku and Imetelstat bind the telomerase RNA (depicted in **Figure S1C**) [23], [41] we tested if they can interfere with each other's binding to hTR. To this end, we performed electrophoretic mobility shift assays (EMSAs). Our results indicate that 1 µM Imetelstat can inhibit the binding of Ku to hTR (**Figure 3A** **)**. As previously reported, slower migrating forms of hTR are observed in the presence of purified Ku (33) (**Figure 3A** **,** compare lane 2 with lane 1). A relatively low concentration (0.5 µM) of Imetelstat inhibited Ku-hTR complex formation (**Figure 3A** **,** lane 3), and a complete loss of complex formation occurred at 1 µM Imetelstat (**Figure 3A**, lane 4). Higher concentrations (2 µM) of a control sense oligonucleotide failed to completely inhibit the formation of the Ku-hTR complex (**Figure 3A**, lanes 7–10). We next assessed whether Ku affects the binding of Imetelstat to hTR. We used 250 pM radiolabeled Imetelstat (**Figure 3B**, lane 1). The addition of 0.02 pmol hTR resulted in the decreased mobility of the labeled Imetelstat (**Figure 3B**, lane 2). Addition of 0.2 and 0.6 µg of Ku resulted in a supershift of the hTR-Imetelstat complex (**Figure 3B**, lanes 3 and 4), suggesting that Ku does not disrupt the binding of Imetelstat to hTR and that the three can exist in a complex in the presence of an excess of Ku relative to the concentration of Imetelstat (1 µM Imetelstat in Figure 3A compared to 250 pM in Figure 3B).

### Targeting either the catalytic site of telomerase or the hTR-Ku interaction sensitizes telomerase positive CLL lymphocytes to FLU

To investigate the contribution of telomerase activity and the Ku-hTR interaction to FLU resistance we utilized a catalytic site inhibitor of telomerase, BIBR1532 [42] and an oligonucleotide complementary to the Ku binding site in hTR (Ku oligo, **Table S1**). We treated twelve telomerase positive samples with FLU alone or in combination with 1 µM Imetelstat, the Ku oligonucleotide (1 µM), or 2.5 µM BIBR1532 [43]. Statistical analysis revealed that the three compounds significantly sensitized primary CLL lymphocytes to FLU (ANOVA, p<0.007) (**Figure 3C**) with median R values of 1.6, 1.8 and 1.2 for Imetelstat, the Ku oligo and BIBR1532 respectively. Thus, both telomerase activity and binding of Ku to hTR might contribute to the survival of primary CLL lymphocytes exposed to FLU.

### Imetelstat increases FLU-induced γ-H2AX in telomerase positive CLL lymphocytes

**Figure 4:**
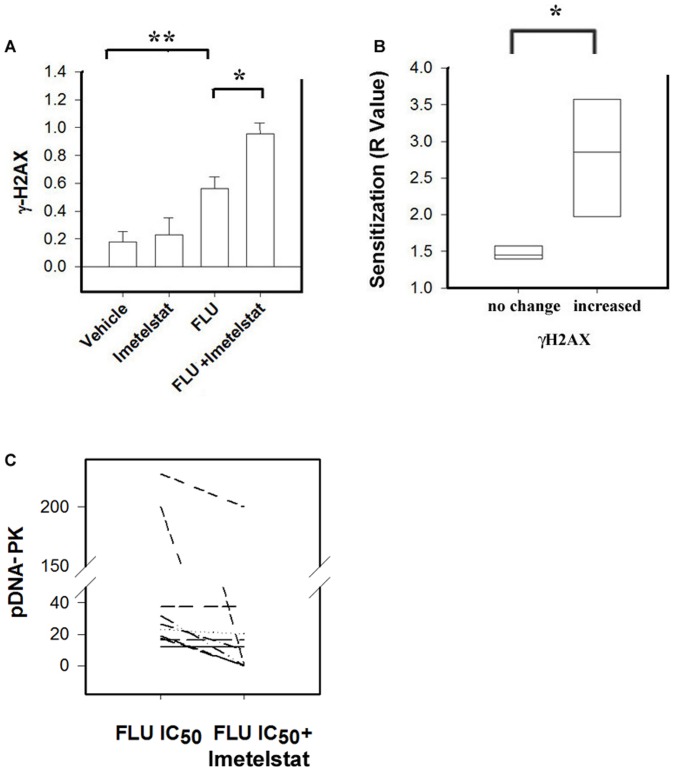
Imetelstat increases FLU-induced γH2AX and decreases FLU-induced pDNA-PK. A) The graph represents the mean levels of γH2AX (phosphorylated H2AX) (y-axis) after treatment of CLL lymphocytes as indicated (x-axis) in eight samples. The whiskers represent the standard error for each group of four samples tested. One Way Analysis of Variance, *p<0.001. B) The median sensitization R values (x-axis) in CLL lymphocyte samples where Imetelstat did not affect FLU-induced γ-H2AX (left bar) was significantly lower than in CLL lymphocyte samples where Imetelstat increased FLU-induced γH2AX (right bar) with respective median values of 1.4 and 1.8 (t-test *p<0.05). C) Changes in DNA-PK serine 2056 (pDNA-PK) in ten primary CLL lymphocyte samples treated with FLU alone (IC_50_) or in combination with 1 µM Imetelstat. The values in the y-axis represent fold change in pDNA-PK with respect to vehicle treated lymphocytes. 1 µM M Imetelstat significantly decreased FLU-induced pDNA-PK (Wilcoxon Signed Rank Test, p = 0.004).

Next we assessed the effects of Imetelstat on the DNA repair capacity of primary CLL lymphocytes exposed to FLU. By western blot analysis we monitored the phosphorylation state of H2AX (γH2AX), an early marker of induced DNA damage, twenty four hours after treatment in eight telomerase positive samples (6, 34). At this time point there was no detectable induction of cell death as monitored by expression of Annexin V (data not shown). Treatment with FLU in combination with 1 µM Imetelstat resulted in a significant 3-fold increase of FLU-inducedγ-H2AX in half of the samples tested compared to vehicle-treated lymphocytes (ANOVA p<0.001) (**Figure 4A**). In the four samples in which Imetelstat did not significantly affect FLU-induced γ-H2AX levels (p>0.05, data not shown) the sensitization was significantly lower than in samples in which Imetelstat increased FLU-induced γ-H2AX levels (median values of 1.4 vs. 2.8, p<0.05 respectively) (**Figure 4B**). Representative γ-H2AX western blots after cells were treated are shown in **Figure S1D**.

### Imetelstat decreases FLU-induced DNA-PK autophosphorylation in telomerase positive CLL lymphocytes

Reports in the literature suggest that expression of DNA-PKcs or its DNA binding subunit, Ku, might be limiting factors in the DNA repair capacity and survival of primary CLL lymphocytes [44] [18]. Inhibition of this process using specific DNA-PK inhibitors sensitizes primary CLL lymphocytes to FLU [33] [18]. To assess the effect of Imetelstat on FLU-induced DNA-PK activation, we quantified the percentage of pDNA-PK positive CLL lymphocytes after treatment with FLU alone or in the presence of 1 µM Imetelstat. Statistical analysis of the results shows that 1 µM Imetlestat inhibited FLU-induced pDNA-PK in seven of the ten samples tested (paired t-test p = 0.004 **Figure 4C**). A representative sample of the analysis performed is shown in **Figure S1E**.

## Discussion

The reported association between FLU resistance and telomerase activity together with the sensitization effect of a catalytic site inhibitor of telomerase (BIBR1532) indicates that telomerase activity can contribute to FLU resistance in primary CLL lymphocytes *in vitro*. Our observations are in agreement with reports demonstrating that telomerase activity can promote DNA repair in both cycling and non cycling cells [10], [12], [13]. Furthermore, it has been recently reported that short term treatment with Imetelstat increases irradiation-induced γ-H2AX and DNA damage in esophageal cancer cells [45]. In this context, we provide evidence that Imetelstat can exacerbate FLU-induced γ-H2AX and modulate DNA-PKcs autophosphorylation upon *in vitro* FLU treatment in primary CLL lymphocytes. Imetelstat-mediated sensitization of lymphocytes from CLL patients to FLU was associated with the basal expression of Ku80. Mutations in either Ku70 or Ku80, in most organisms examined, results in the expected deficits in DNA DSB repair, but in human cells a Ku deletion (but not a DNA-PKcs deletion) is lethal suggesting that Ku may have functions independent of NHEJ [24], [25]. The Ku70/80 heterodimer has been shown to mediate cell-cell and cell-extracellular matrix adhesion in different cell types [46]. Although Ku is highly expressed in established human cancer cell lines, the expression of this heterodimer is variable in normal and malignant human tissues [47], [48]. In particular, in CLL lymphocytes Ku mRNA levels are lower when compared to normal B cells [26]. In agreement with these reports, we find an eighteen-fold difference in the expression of Ku80 among the primary CLL samples tested.

Although Imetelstat-mediated sensitization to FLU was not directly associated with telomerase activity (or drug-induced changes in telomerase activity), the association between Imetelstat-mediated sensitization to FLU and Ku80 expression was stronger in primary CLL lymphocytes with detectable telomerase activity. The biological relevance of this observation in the context of DNA damage induction in quiescent cells is not known, however, a recent study found that Imetelstat treatment leads to the disappearance of hTR localization at telomeres and accumulation of hTR in Cajal bodies [49]. In concert with a telomerase-associated protein, TCAB1, the CAB box within hTR regulates its localization to the Cajal bodies [50]. We speculate that Ku could facilitate hTR trafficking and/or assembly of telomerase since the Ku binding site of hTR (targeted by the Ku oligo) comprises the CAB box (**Table S2**). In agreement with this hypothesis, the EMSA experiments indicate that Imetelstat can interfere with the binding of Ku to hTR *in vitro*, and may do so in CLL lymphocytes treated with FLU. The biological importance of the interaction of Ku with hTR is highlighted by our results showing that the Ku oligo sensitized primary CLL lymphocytes to FLU. Thus, Imetelstat could affect telomerase directly by binding to hTR and indirectly by affecting telomerase biogenesis by decreasing Ku binding to hTR. Since in the presence of excess Ku, Imetelstat did not abolish the binding of Ku to hTR, the effect of Imetelstat on the Ku-hTR interaction may be Ku70/80 concentration dependent. Thus, it is possible that in CLL lymphocytes, Ku expression is a rate limiting factor in several biological processes that contribute to overcome FLU cytotoxicity (i.e recruitment of DNA-PK to DSBs, modulation of DNA repair and binding of Ku to hTR). In addition, as proposed by others, Ku could contribute to the protection of telomeres [24], [25].

Interestingly a few studies have reported rapid effects of Imetelstat linked to changes in cell morphology due to effects on cell adhesion [29], [51]. In a different study, short term Imetelstat treatment inhibited clonogenic multiple myeloma growth associated with cancer stem cell differentiation [51]. The short term effect was associated with decreased expression of genes typically expressed by stem cells. These reports suggest that Imetelstat, by mechanisms perhaps unrelated to its effects on telomerase, can affect cancer cell survival. This telomerase-independent mechanism could explain the effect of Imetesltat on FLU resistance in CLL lymphocyte samples with undetectable telomerase activity.

## Conclusions

Imetelstat sensitizes quiescent, non-dividing primary CLL lymphocytes to FLU cytotoxicity *in vitro* by interfering with pro-survival processes which are both dependent and independent of telomerase activity, but independent of DNA synthesis at telomeres. Although the mechanisms involved in Imetelstat-mediated sensitization to FLU in primary CLL lymphocytes remain speculative, the effect occurs in lymphocytes from clinically resistant patients and/or cytogenetic abnormalities (i.e. del17 and del11) that are associated with bad prognosis in CLL [52] [1]. In addition, our results suggest that there is a functional cross talk between the RNA component of telomerase and Ku and highlight a role for the telomerase RNA in the regulation of the response to FLU in primary CLL lymphocytes. Clinically, our results identify telomerase as a possible target to increase the cytotoxicity and the efficacy of FLU to reduce tumor burden in CLL patients, including clinically resistant cases and/or positive for unfavourable prognostic factors such as U-IgVH, del11 and del17.

## Supporting Information

Figure S1 A) The box represents the 25^th^/75^th^ percentiles and the line in the box represents the median value of FLU IC_50_ (y-axis). The median FLU IC_50_ was significantly higher in the lymphocytes from clinically resistant (CR, n = 7) patients when compared to the clinically untreated (U, n = 21) and clinically treated patients (T, n = 11) (ANOVA p<0.05). Further Mann-Whitney Rank Sum Test indicates that lymphocytes from clinically resistant patients are more resistant to FLU than lymphocytes from clinically untreated patients (*p = 0.005). B) Ku80 expression was assessed using 10 µg of protein extracts from lymphocytes from twenty four CLL patients by western blot as described in materials and methods. The Ku80 values obtained were normalizedusing the values obtained after reprobing for α-actin. C) Schematic depicting hTR and the mapped binding sites of Imetelstat and Ku70/80. D) Representative western blots using 20 µg of protein extracts from lymphocytes twenty four hours after *in vitro* treatment as indicated, and showing that Imetelstat increased FLU-induced γH2AX. Equal loading was assessed by reprobing for Ku80. E) Representative analysis of the effect of 1 µM Imetelstat on FLU-induced DNA-PK autophosphorylation of CLL lymphocytes treated with vehicle (upper panel), the FLU IC_50_ concetration (middle pannel) or the combination of FLU IC_50_ and 1 µM Imetelstat (bottom panel). The x-axis represents pDNA-PK staining and the y-axis the number of positive cells.
(TIF)

Table S1 **Characteristics of CLL cases and patient samples in vitro.** The characteristics of the cases, clinical status of the patients, Rai Stage, age, peripheral lymphocytes counts and CD38 were obtained from the Hematology Clinic at the Jewish General Hospital. The cut off value between unmutated and mutated IgVH cases is 2% with respect to germline. Del11 and del17 status were assessed at the Jewish General Hospital Molecular Pathology Laboratory using standard techniques. Clinical Status, U: Untreated, T: Treated, R: Resistant. TL activity, Telomerase Activity. R Sensitization, R value  =  FLU IC_50_/IC_50_ of FLU+ 1 µM Imetelstat. IgVH Status, U: unmutated IgVH, M: mutated IgVH. NA, not available. The superscripts indicate the clinical treatment received by the patients. a: Chlorambucil (CLB). b: Cytoxan. c: FLU. d:Rituximab.
(TIF)

Table S2 **Oligonucleotides Targetting hTR-Ku binding region.** nt: nucleotides. Underlined sequences are predicted single-stranded regions according to proposed secondary structures of telomerase RNA as determined by phylogenetic comparative analysis performed before [Chen, J. L., M. A. Blasco, et****al. (2000)]. Bold sequence denotes the CAB box.
(TIF)
