# Supplementary figures and images for: Telomerase Contributes to Fludarabine Resistance in Primary Human Leukemic Lymphocytes
Source: PLoS One. 2013 Jul 29;8(7):e70428. doi: 10.1371/journal.pone.0070428 (PMC3726637; doi:10.1371/journal.pone.0070428)

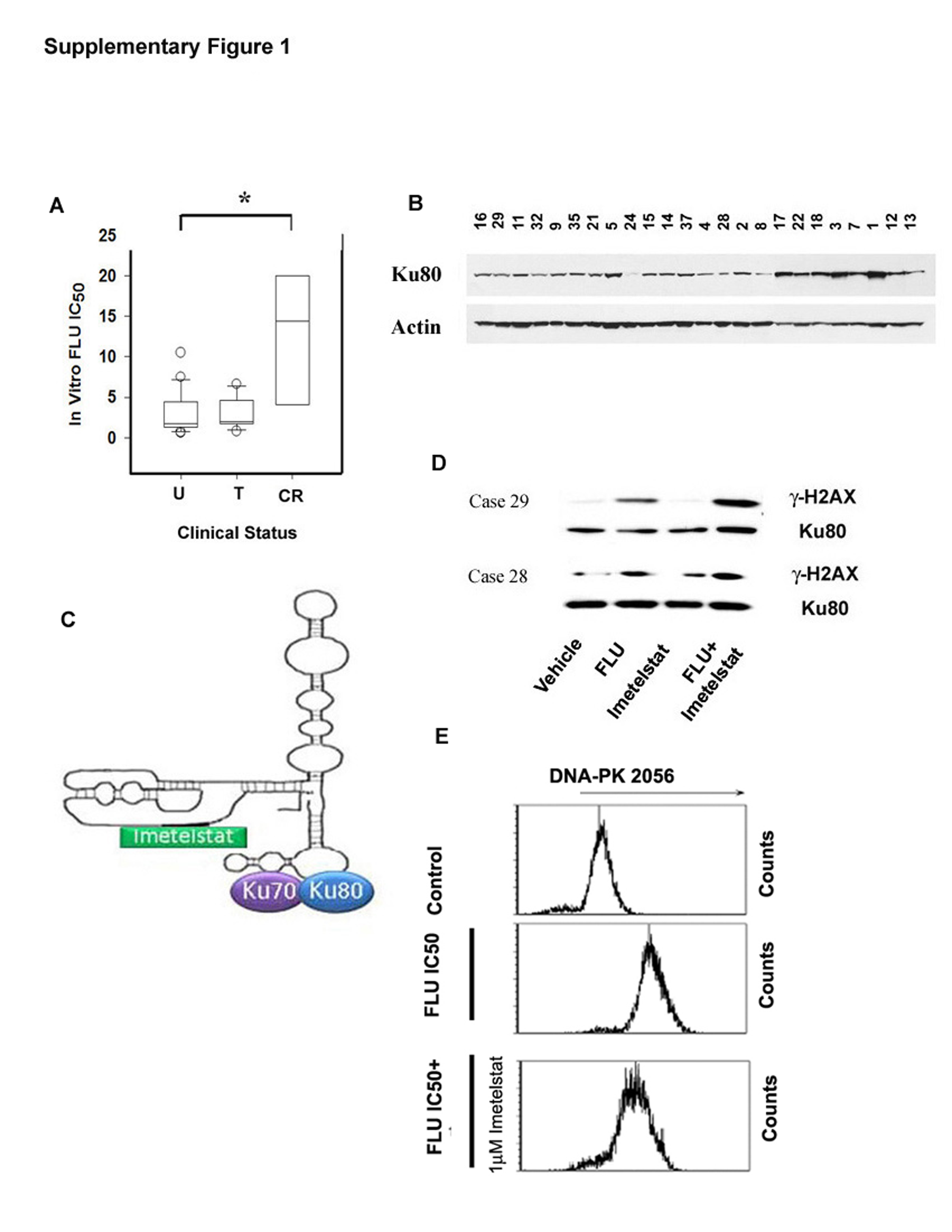

Supplement: Figure S1 — A) The box represents the 25th/75th percentiles and the line in the box represents the median value of FLU IC50 (y-axis). The median FLU IC50 was significantly higher in the lymphocytes from clinically resistant (CR, n = 7) patients when compared to the clinically untreated (U, n = 21) and clinically treated patients (T, n = 11) (ANOVA p<0.05). Further Mann-Whitney Rank Sum Test indicates that lymphocytes from clinically resistant patients are more resistant to FLU than lymphocytes from clinically untreated patients (*p = 0.005). B) Ku80 expression was assessed using 10 µg of protein extracts from lymphocytes from twenty four CLL patients by western blot as described in materials and methods. The Ku80 values obtained were normalizedusing the values obtained after reprobing for α-actin. C) Schematic depicting hTR and the mapped binding sites of Imetelstat and Ku70/80. D) Representative western blots using 20 µg of protein extracts from lymphocytes twenty four hours after in vitro treatment as indicated, and showing that Imetelstat increased FLU-induced γH2AX. Equal loading was assessed by reprobing for Ku80. E) Representative analysis of the effect of 1 µM Imetelstat on FLU-induced DNA-PK autophosphorylation of CLL lymphocytes treated with vehicle (upper panel), the FLU IC50 concetration (middle pannel) or the combination of FLU IC50 and 1 µM Imetelstat (bottom panel). The x-axis represents pDNA-PK staining and the y-axis the number of positive cells. (TIF) [file pone.0070428.s001.tif]

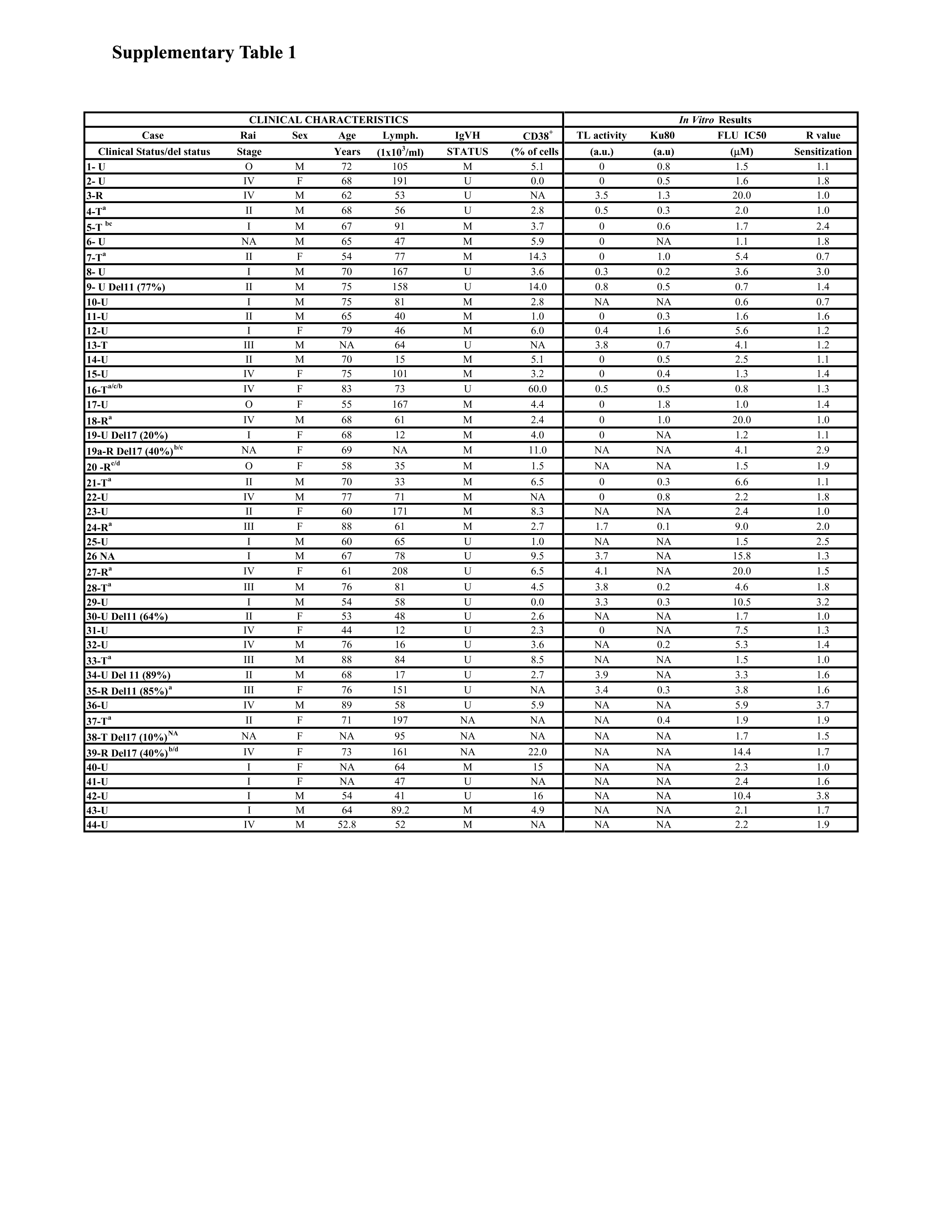

Supplement: Table S1 — Characteristics of CLL cases and patient samples in vitro. The characteristics of the cases, clinical status of the patients, Rai Stage, age, peripheral lymphocytes counts and CD38 were obtained from the Hematology Clinic at the Jewish General Hospital. The cut off value between unmutated and mutated IgVH cases is 2% with respect to germline. Del11 and del17 status were assessed at the Jewish General Hospital Molecular Pathology Laboratory using standard techniques. Clinical Status, U: Untreated, T: Treated, R: Resistant. TL activity, Telomerase Activity. R Sensitization, R value = FLU IC50/IC50 of FLU+ 1 µM Imetelstat. IgVH Status, U: unmutated IgVH, M: mutated IgVH. NA, not available. The superscripts indicate the clinical treatment received by the patients. a: Chlorambucil (CLB). b: Cytoxan. c: FLU. d:Rituximab. (TIF) [file pone.0070428.s002.tif]

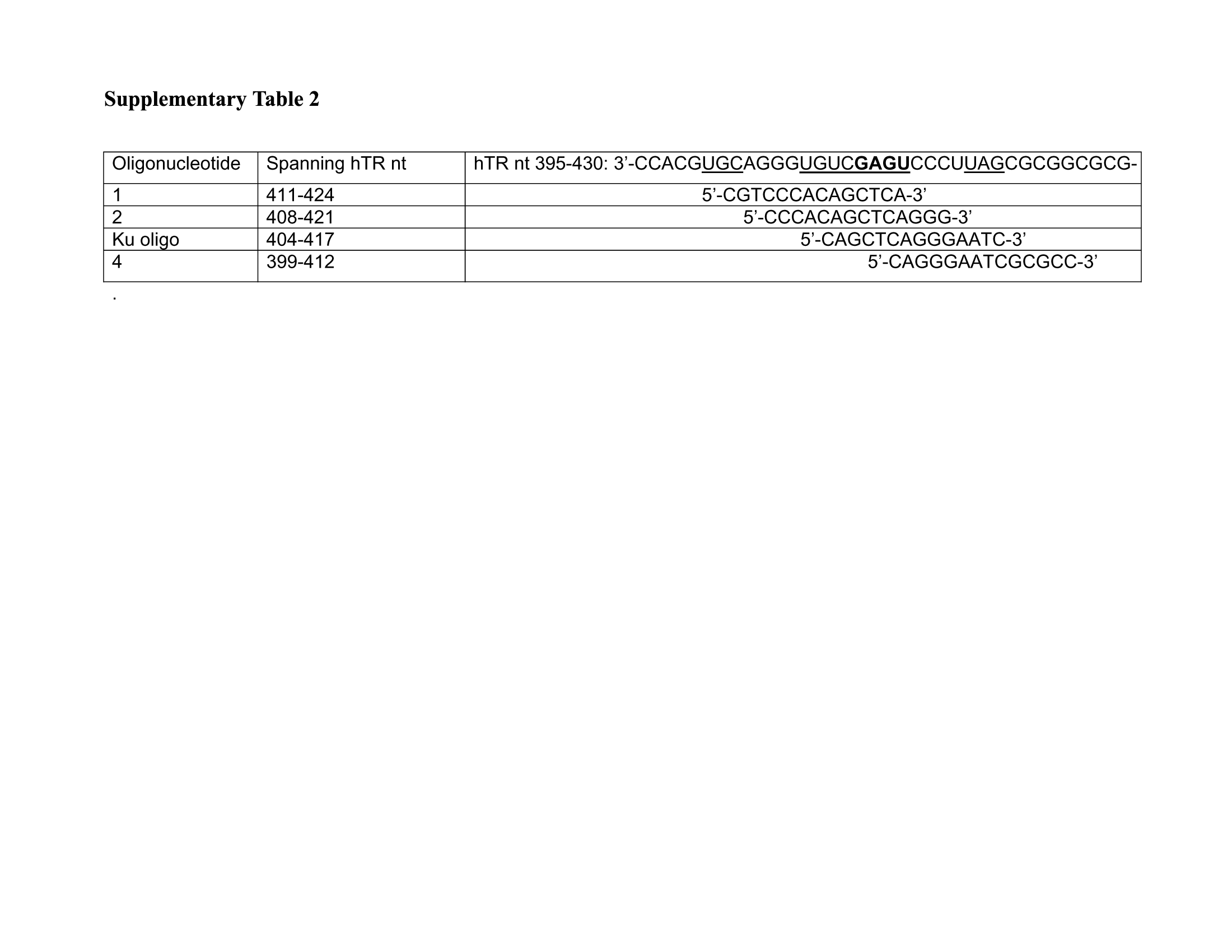

Supplement: Table S2 — Oligonucleotides Targetting hTR-Ku binding region. nt: nucleotides. Underlined sequences are predicted single-stranded regions according to proposed secondary structures of telomerase RNA as determined by phylogenetic comparative analysis performed before [Chen, J. L., M. A. Blasco, etal. (2000)]. Bold sequence denotes the CAB box. (TIF) [file pone.0070428.s003.tif]
